# Evolution of respiratory syncytial virus genotype BA in Kilifi, Kenya, 15 years on

**DOI:** 10.1038/s41598-020-78234-0

**Published:** 2020-12-03

**Authors:** Everlyn Kamau, James R. Otieno, Clement S. Lewa, Anthony Mwema, Nickson Murunga, D. James Nokes, Charles N. Agoti

**Affiliations:** 1 Epidemiology and Demography Department, Kenya Medical Research Institute (KEMRI) – Wellcome Trust Research Programme, Kilifi, Kenya; 2 School of Life Sciences and Zeeman Institute (SBIDER), University of Warwick, Coventry, UK; 3 School of Health and Human Sciences, Pwani University, Kilifi, Kenya; 4 Present Address: Nuffield Department of Medicine, University of Oxford, Oxford, UK; 5 Present Address: Fogarty International Center, NIH, Bethesda, MD USA

**Keywords:** Infectious diseases, Respiratory tract diseases, Molecular evolution

## Abstract

Respiratory syncytial virus (RSV) is recognised as a leading cause of severe acute respiratory disease and deaths among infants and vulnerable adults. Clinical RSV isolates can be divided into several known genotypes. RSV genotype BA, characterised by a 60-nucleotide duplication in the G glycoprotein gene, emerged in 1999 and quickly disseminated globally replacing other RSV group B genotypes. Continual molecular epidemiology is critical to understand the evolutionary processes maintaining the success of the BA viruses. We analysed 735 G gene sequences from samples collected from paediatric patients in Kilifi, Kenya, between 2003 and 2017. The virus population comprised of several genetically distinct variants (*n* = 56) co-circulating within and between epidemics. In addition, there was consistent seasonal fluctuations in relative genetic diversity. Amino acid changes increasingly accumulated over the surveillance period including two residues (N178S and Q180R) that mapped to monoclonal antibody 2D10 epitopes, as well as addition of putative N-glycosylation sequons. Further, switching and toggling of amino acids within and between epidemics was observed. On a global phylogeny, the BA viruses from different countries form geographically isolated clusters suggesting substantial localized variants. This study offers insights into longitudinal population dynamics of a globally endemic RSV genotype within a discrete location.

## Introduction

Respiratory syncytial virus (RSV) is a leading cause of severe lower respiratory tract infections, with an estimated 3.2 million annual hospitalizations and approximately 60,000 deaths globally, primarily in children younger than 1-year-old^1–3^. Among adults, RSV produces a wide range of clinical symptoms including upper respiratory tract infections, severe lower respiratory tract infections, and exacerbations of underlying disease^4,5^. RSV epidemics exhibit clear patterns of seasonality and repeat infections are common throughout life^6^. Despite its global health impact, effective RSV prophylactics are limited, but multiple vaccine candidates and monoclonal antibodies are in different stages of development and licensure^7,8^.

RSV genome, a negative-sense single-stranded RNA molecule, encodes 11 proteins including the fusion (F) and attachment (G) glycoproteins, which mediate virus binding and entry into host cells and are targets for neutralizing antibodies^9^. Two groups, RSVA and RSVB, defined based on antigenic and genetic variability within the glycoproteins co-circulate worldwide, causing seasonal epidemics^10,11^. Several RSV genotypes, 10 for RSVA and 13 for RSVB, have been identified to date^12,13^. In 1999, the RSVB genotype BA emerged with a 60-nucleotide duplication in the G gene and disseminated globally, replacing all other RSVB genotypes^14,15^. It is likely the additional residues of the 60-nucleotide duplication provide a selective advantage and modified the antigenic characteristics of the G protein, allowing escape from antibody neutralization, as well as enhancing fitness^15^.

RSV longitudinal surveillance studies in Kilifi, coastal Kenya, have revealed a high disease burden particularly in infancy and early childhood^4,16–23^. Studying the genetic and antigenic evolution of local strains is crucial for understanding how annual RSV epidemics are maintained locally. This may help with designing comprehensive infection reduction/control measures and rationalize respiratory virus epidemic response policies^24,25^. Studies of the origins of RSV seed strains for the local regular epidemics, hubs of infection transmission, spread patterns, viral evolution and reinfection patterns would lead to better epidemic management^26^ as recently demonstrated for emerging viruses like Ebola, Zika, SARS-CoV, SARS-CoV-2, Influenza A/H1N1/09)^27,28^.

The RSVB genotype BA was first detected in Kilifi in early 2003 and became dominant from 2004 onwards except for sporadic detection of the genotype SAB4 between the years 2011 and 2013^24^. We analyzed the G glycoprotein of RSVB genotype BA strains collected over 15 successive RSV epidemics in Kilifi. We show that locally, the genotype is characterized by discrete temporal genetic clustering and sequential variant replacement between and sometimes within epidemics. This work extends previous analyses^18,24^ by a further six years of new data and augments our knowledge of the evolutionary trajectory and adaptation of a single RSV genotype in a single local setting when observed longitudinally.

## Results

Over 15 RSV epidemics (2002 to 2017), a total of 903/12203 (7.4%) respiratory samples tested positive for RSVB. G gene sequencing was attempted for all RSVB positive samples and was successful for 857 (95%) RSVB positive samples. The proportion of RSVA and RSVB samples in each epidemic ranged from 3.8 to 77.0% (Table 1). RSVB dominated the 2004/5, 2007/8, 2011/12 and 2016/17 epidemics and co-dominated with RSVA in the 2009/10 and 2014/15 epidemics (Table 1). The annual distribution of RSV cases by age from 2002 to 2017 are shown in Supplementary Table 1. Although the hospital-based RSV surveillance in Kilifi was established in 2002, the genotype BA was not detected until January 2003. From the RSVB positive samples, RSVB BA viruses were confirmed during sequence assembly and analysis by the presence of a 60-nucleotide duplication in the C-terminal of the G gene. Overall, a total of 735 genotype BA G gene sequences spanning nucleotides 214 to 981 of the RSVB strain B1 (DQ227363) were obtained.

**Table 1:** Number of RSVB positive samples and genotype BA sequences by epidemic in Kilifi, 2002 to 2017.

RSV epidemic	RSV positive samples	Number (%) of RSV-B samples^a^	RSVB genotype BA sequences	Number (%) of RSVA samples^a^
2002/3	89	35 (39.3)	4	42 (47.2)
2003/4	114	16 (14)	6	68 (59.6)
2004/5	183	**119 (65)**	152	46 (25.1)
2005/6	239	9 (3.8)	9	224 (93.7)
2006/7	195	22 (11.3)	22	153 (78.5)
2007/8	256	**197 (77)**	141	33 (12.9)
2008/9	208	41 (19.7)	14	154 (74)
2009/10	259	95 (36.7)	58	120 (46.3)
2010/11	279	21 (7.5)	17	241 (86.4)
2011/12	161	**102 (63.4)**	81	55 (34.2)
2012/13	152	27 (17.8)	14	110 (72.4)
2013/14	117	42 (35.9)	42	75 (64.1)
2014/15	202	69 (34.2)	53	133 (65.8)
2015/16	149	83 (55.7)	72	66 (44.3)
2016/17	76	**53 (69.7)**	50	23 (30.3)

Bold values indicate that the proportion of RSV-B samples was greater than RSV-A samples.

^a^Percentage of RSV positive samples.

### Genetic diversity of the genotype BA

**Figure 1:**
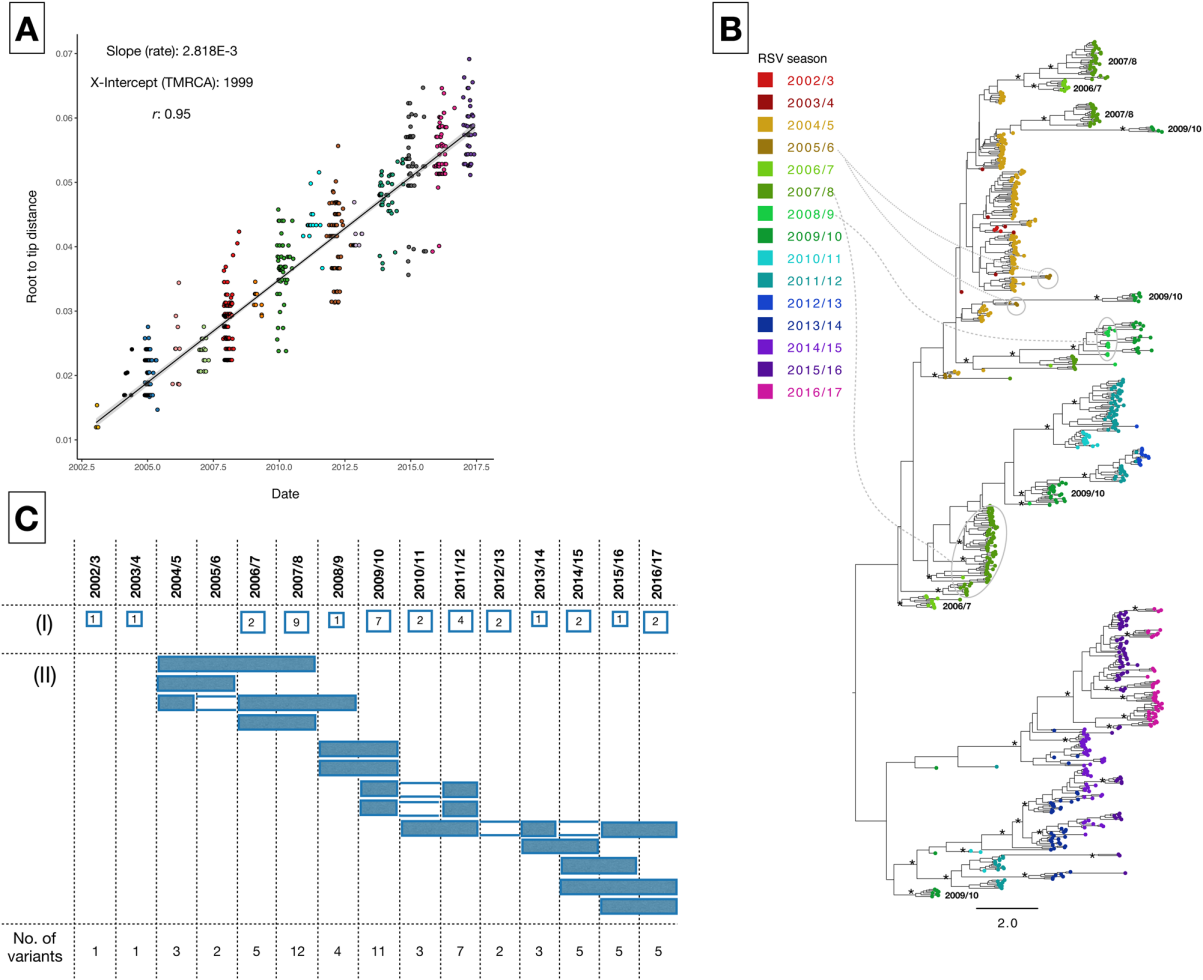
(**A**) Correlation plot of root-to-tip genetic distance against sampling date for a phylogeny estimated from RSVB genotype BA 735 G gene sequences sampled from Kilifi, Kenya. The estimated correlation coefficient and R^2^ values were 0.95 and 0.9, respectively. (**B**) Maximum clade credibility (MCC) tree inferred for 735 G gene sequences (756 nucleotides) from Kilifi, with tip labels colored by RSV epidemic. Node support is indicated by (*) for posterior probabilities > 0.9. (**C**) Temporal occurrence of the 56 RSVB genetic variants (rows) identified in Kilifi between 2003 and 2017. A variant was defined as a virus or group of viruses with ≥ 4 nucleotide differences in the sequenced G region (see “Methods” section). The number of variants circulating only in a single epidemic are shown in (I) and variants that circulated in more than one epidemic are indicated by filled rectangles in (II).

The sequenced G gene region showed 3% nucleotide divergence (overall mean *P* distance) over the entire period and varied by a maximum of 38 nucleotides within an epidemic. Genetic divergence increased proportionally with time, Fig. 1A, indicating clock-like evolution. Molecular clock phylogenies showed a well-ordered diversification of the genotype BA since its introduction in Kilifi (Fig. 1B). Viruses from the same epidemic formed multiple phylogenetic clusters and often, sequences from the preceding epidemic were often positioned at the basal nodes of those in the successive epidemic, suggesting sequential virus circulation and persistence between epidemics. A total of 56 distinct variants were identified, some were singletons suggesting either low level transmission or unsampled genetic diversity. The 2007/8 and 2009/10 epidemics were the most heterogeneous, with 12 and 11 variants, respectively. Up to four variants circulated over multiple epidemics (Fig. 1C), and some were observed in non-consecutive epidemics likely indicating re-introduction of variants that had been previously observed locally.

### Evolutionary history of the genotype BA in Kilifi

**Figure 2:**
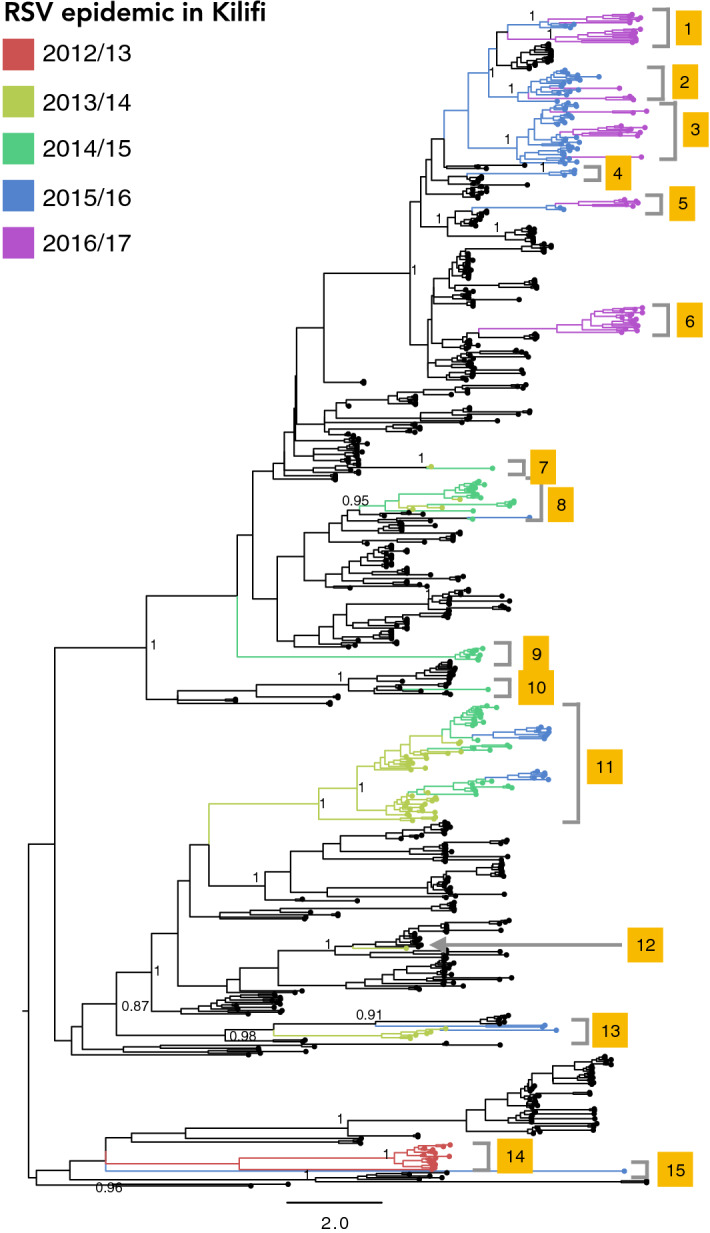
Maximum likelihood phylogeny estimated from 716 genotype BA G gene sequences sampled globally between 2012 and 2017. Discrete virus introductions in Kilifi are marked on the phylogeny. Terminal branches with sequences from Kilifi are coloured by RSV epidemic. Terminal branches with black circles are indicate sequences from locations outside Kilifi. Clade posterior probabilities are shown for selected nodes (> 0.75).

Our previous study reported the number of virus introductions into Kilifi from 2002/3 to 2011/12 epidemics^24^. To place the Kilifi genotype BA viruses in the context of global RSVB diversity, we analyzed the 2012/13 to 2016/17 Kilifi sequences (*n* = 233) and 583 global sequences sampled from 16 countries between 2012 and 2017. In the current study, distinct geographical clusters were evident with viruses clustering primarily by country of origin. The 2012/13 to 2016/17 RSVB epidemics in Kilifi were seeded by at least 15 virus introductions (Fig. 2). For many countries, viruses circulating during the same year were not placed into single monophyletic groups but in multiple clusters of assorted sizes (Fig. 2), indicative of multiple virus entries followed by local spread and genetic expansion. Between 2012 and 2017, several other variants circulated outside Kilifi suggesting that contemporaneous RSV epidemics are as a result of different localized variants and less likely sequential transmission between countries.

### Virus population dynamics

**Figure 3:**
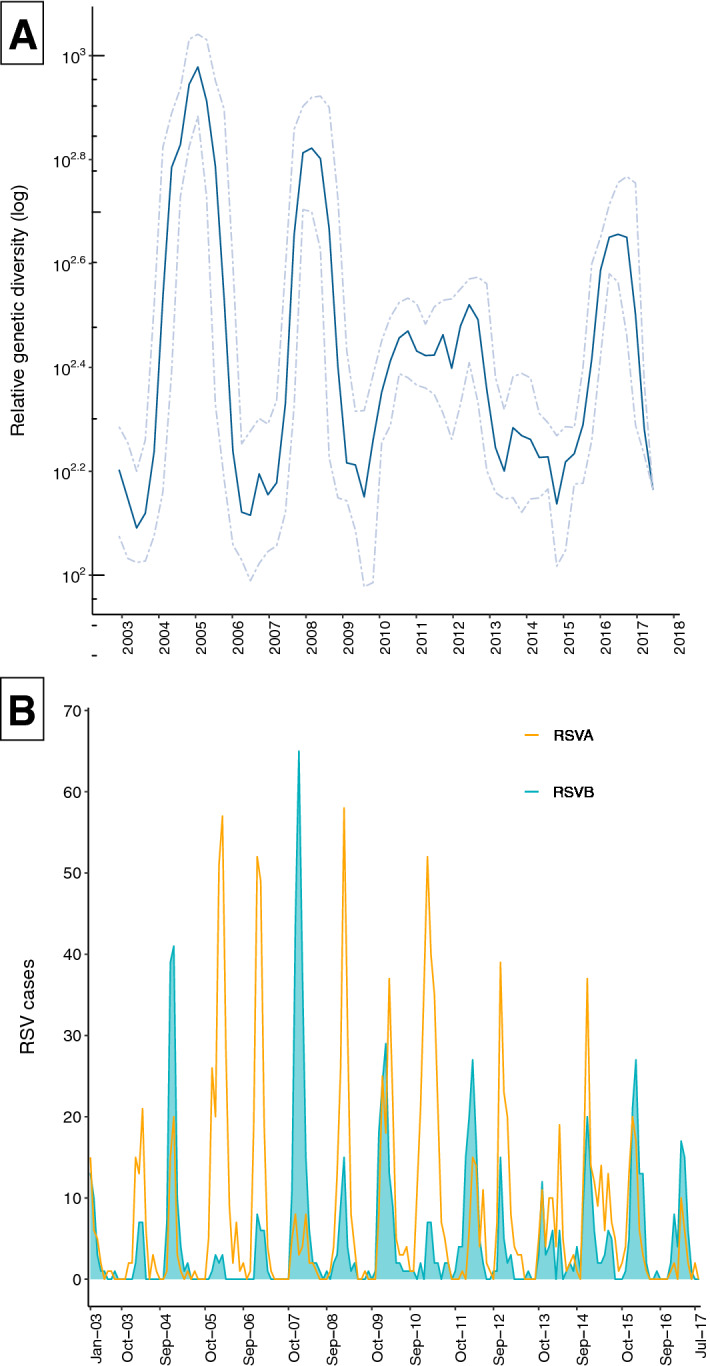
(**A**) Relative genetic diversity estimated using the Gaussian Markov Random Field (GMRF) Bayesian skyride coalescent model. Solid lines represent mean relative genetic diversity while the dotted lines indicate the 95% HPD intervals. (**B**) Epidemic patterns of RSV antigenic groups (RSVA and RSVB) in Kilifi, Kenya, from 2002 to 2017.

We used Bayesian skyride analysis to estimate temporal changes in relative genetic diversity (Fig. 3A). Elevation in relative genetic diversity was correlated with increased RSVB activity and appearance of new viral populations in the 2004/5, 2007/8, and 2016/17 epidemics (Fig. 3B). Demographic expansions were interspersed by constrictions in the effective population size for example in 2006, 2009/10, and 2014/15, characteristic of bottleneck effects and variant replacement between epidemics^15,29^. It was previously suggested that the expansion in the effective population in 2005 coincided with the predominance and rapid dissemination of the genotype BA, relative to the rest of the group B viruses^15^. The increase in relative genetic diversity between 2006 and 2008 could also be related to the introduction and dissemination of viruses with novel mutations in 2007/8 as described below.

### Amino acid changes

**Figure 4:**
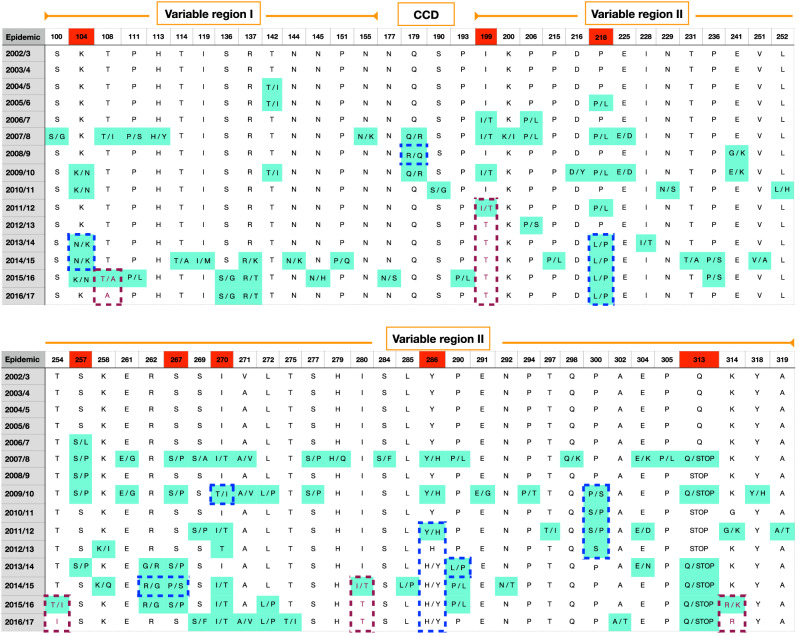
Amino acid differences in the G protein relative to majority consensus residue for each position indicated. Shown are the differences or amino acid variants detected in more than 10% of the viruses. Red dotted borders indicate new and possibly beneficial variants that emerged and became fixed in the viral population. Blue dotted borders indicate amino acid switch from ‘minority’ to ‘majority’ variant.

Numerous amino acid (aa) changes occurred with variable frequencies over the 15 epidemics, and commonly these changes involved reversal or ‘toggling’ between codons within or between epidemics. Codon positions exhibiting frequent (> 10% of sequences) reversals are marked in red in Fig. 4. The 2002/3 to 2006/7 epidemics had few changes, while the 2007/8 epidemic saw increased RSVB activity and appearance of several new amino acid changes. Additional changes emerged in every epidemic after 2007/8 particularly in RSV seasons with high RSVB incidence. Several positions had more than one amino acid change, including T108I/A, R137K/T, P206L/S, E241G/K, S257L/P, K258I/Q, S269A/P/F, E304K/D/N and K314G/R.

Occasionally, new and possibly beneficial variants emerged and became fixed in the viral population (red dotted borders, Fig. 4). For example, the I199T amino acid substitution was introduced in 2011/12 and Threonine (T) became fixed at position 199 in the subsequent epidemics, while the T108A, T254I, I280T and R314K substitutions became fixed in the 2015/16 and 2016/17 epidemics. We observed amino acid switch from ‘minority’ to ‘majority’ variant: when a ‘minor’ amino acid variant (< 30% frequency) occurred in the majority (> 70%) of viruses in the successive epidemic(s) or re-appeared in the population after absence in preceding epidemic(s) (blue dotted borders, Fig. 4). For instance, Leucine (position 218) reappeared in 2013/14 and became dominant through to 2016/17 epidemic. This is also evident for Histidine at position 286. The emergence of viruses with Q313Stop mutation occurred in 2007/8 akin to a previous report^15^, then disappeared in 2008/9, reappeared in 2009/10, and disappeared again until 2013/14. This codon has been proposed to be under positive selection^30^.

**Figure 5:**
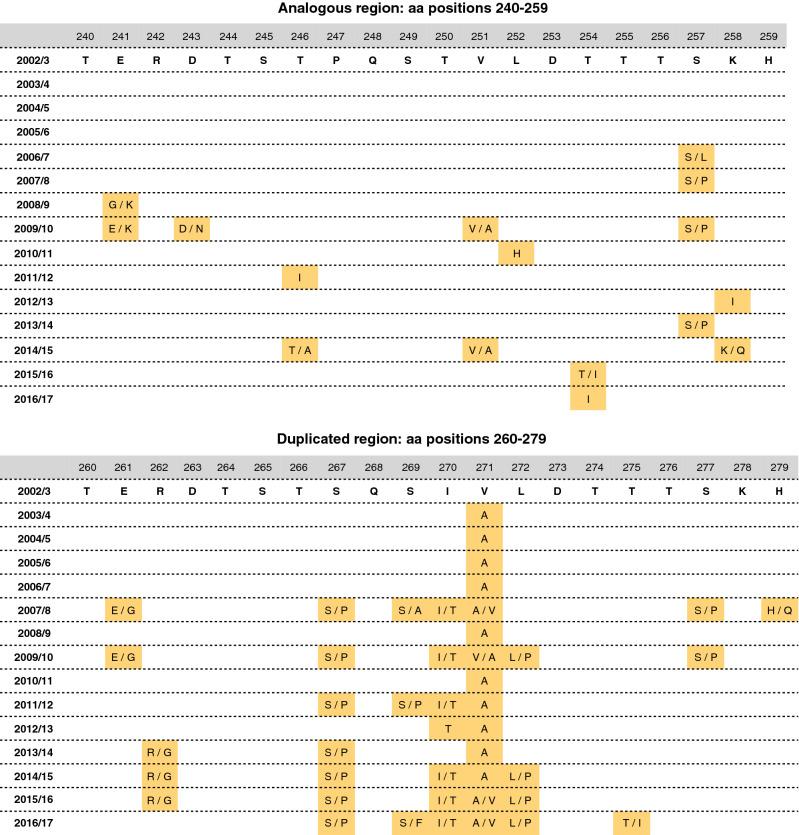
Amino acid changes in the 60-nt (20-aa) duplication region relative to the earliest RSVB genotype BA sequences in the Kilifi dataset.

We also examined variability at the 60-nucleotide analogous (aa 241–259) and duplicated segments (aa 260–279) (Fig. 5). Relative to oldest BA sequences (2002/3) in Kilifi, the 2003/4 to 2005/6 viruses were nearly identical in both segments. Amino acid changes gradually accumulated in the duplicated segment from the 2007/8 epidemic.

### Sequence variations at the central conserved domain

Between the two mucin-like regions of RSV G is an un-glycosylated central conserved domain (CCD) (positions ~ 155 to 206), with a stretch of 13 aa (164–176) that is strictly maintained in all strains, and four invariant cysteines (residues 173, 176, 182 and 186) with a 1–4, 2–3 disulfide topology forming a cysteine noose that are also highly conserved^31,32^. The third and fourth cysteines (Cys^182^–Cys^186^) form a CX3C motif poised for interaction with CX3CR1, mediating RSV attachment during infection^31^. Human broadly neutralising monoclonal antibodies (mAb) have been reported to bind in the CCD^33–35^.

We found two amino acid substitutions (N178S and Q180R) located within the cysteine noose. The N178S substitution was detected in 14 sequences in the 2014/15 epidemic, while the Q180R mutation appeared in 2007/8 and persisted through to 2009/10. Importantly, Arginine at position 180 was detected or fixed in all the strains sampled in 2008/9. These aa changes are positioned within the antigenic site γ2, the conformational epitope (residues 177–188) of the high affinity broadly neutralising human mAb 2D10^33^. Structurally, the two residues are oriented outward, residue 178 is visible in a loop connecting two alpha-helices and residue 180 occurs in an alpha helix. The two substitutions are relatively conservative (polar, hydrophilic) and unlikely to have a substantial effect on G structure or function but could be molecular adaptations to natural selection pressure.

### N-linked glycosylation

We identified N-linked glycosylation sequons at 14 residues (81, 86, 117, 134, 144, 230, 243, 263, 265, 273, 293, 296, 305 and 310) occurring at varied frequencies (Table 2). The number of N-glycosylation sites increased continuously over the epidemics. The minimum number of possible N-glycosylation sites per sequence was three, and the maximum was four. Two putative N-glycosylation sites (296 and 310) were dominant in the majority (93.2% and 98.1%, respectively) of the sequenced viruses. Amino acid substitutions (N296K/D and S297P), and T312N/A/I, occurring between 2010/11 and 2013/14 resulted in loss of potential N-glycosylation sequons at position 296 and 310, respectively.

**Table 2:** N-linked glycosylation sites and patterns and their occurrence in the RSV-B epidemics in Kilifi, Kenya.

Pattern	Codon position												Epidemics observed
	81	86	127	134	230	243	263	265	273	293	296	310	
1	−	−	−	−	−	−	−	−	−	−	+	+	2002/3, 2003/4, 2004/5, 2005/6, 2006/7, 2007/8, 2008/9, 2009/10, 2010/11, 2011/12, 2012/13, 2013/14, 2014/15
2	−	−	−	−	−	−	−	−	−	+	+	+	2009/10
3	−	−	−	−	−	+	−	−	−	−	+	+	2009/10
4	−	−	+	−	−	−	−	−	−	−	+	+	2007/8
5	−	−	−	−	+	−	−	−	−	−	+	+	2014/15
6	−	−	−	+	−	−	−	−	−	−	+	+	2006/7
7	−	−	−	−	−	−	−	+	−	−	+	+	2004/5
8	−	−	−	−	−	−	+	−	−	−	+	+	2009/10
9	−	−	−	−	−	−	−	−	+	−	+	+	2004/5
10	+	+	−	−	−	−	−	−	−	−	+	+	2014/15, 2015/16,2016/17
Frequency (%)	17.5	17.5	0.4	0.01	0.01	0.7	0.01	0.01	0.01	1	93.7	98.6	

Notably, two new putative N-glycosylation sequons (positions 81 and 86) emerged in 2014/15 and persisted in all the viruses sampled the succeeding epidemics. Changes in glycosylation state can be a source of antigenic novelty conferring selective advantage by ‘masking’ epitopes from antibody recognition. It was observed that addition of glycosylation sites to hemagglutinin in Influenza A can reduce or abolish binding by monoclonal antibodies or human antisera^36^.

### Selection analyses

Evidence for pervasive and episodic selection was found in 13 codon positions: 144, 151, 159, 223, 227, 270, 281, 287, 289, 290, 305, 306, 313. Eight of the 13 positions (underlined) were identified by more than one method. The MEME method found positions 281, 305 and 313 under episodic adaptive selection (occurring heterogeneously across branches), while the FUBAR and FEL methods identified pervasive selection in positions 159 and 306, respectively. The SLAC method estimated the overall mean nonsynonymous/synonymous (d_N_/d_S_) substitution rate ratio as 0.56. Generally, the analyzed G region is under purifying/negative selection (d_N_/d_S_ < 1), and only a very restricted number of codon positions have evolved at a higher non-synonymous substitution rate (> 1, in Supplementary Figure S1).

## Discussion

This study documents the natural history of the RSVB genotype BA viruses in Kilifi, Kenya, over 15 epidemics. Our analysis found substantial genetic diversity exhibited by multiple variants co-circulating within and between epidemics. Shifts in the predominating strains have been suggested to confer a selective advantage by variations in strain- or clade-specific immunity that favors circulation of heterologous variants^37,38^. Virus sequences from successive epidemics clustered together, suggesting variant persistence between RSV seasons. As previously implied^39^, it is possible that genetically close viruses are re-imported each year from unsampled locations rather than persisting locally.

The demographic expansions around 2005 and 2008 denote periods of optimal adaptation for virus replication and widespread dissemination, congruent with proliferation of new lineages less susceptible to immune responses^40^, and were previously described in relation to the global spread of the BA-IV lineage viruses^15^. Fluctuation in relative genetic diversity around 2003 and early 2007 is characteristic of bottleneck effects or ‘viral eclipse phases’ preceding population expansion, probably imposed by selection pressure or as a result of decline of circulating variants^40^.

The 60-nucleotide duplication region showed further accumulation of nucleotide substitutions further than previously reported^15^, which likely points to enhanced fitness and molecular adaptation providing evolutionary advantage of the BA genotype^41^ compared to other RSVB genotypes. Accumulation of nucleotide changes in RSV G gene was also attributed to antibody selection to some extent, as well as selective constraints other than immune selection, for instance, high mutation rate, protein malleability and bottleneck effects^42^.

We observed amino acid reversion patterns in the Kilifi genotype BA viruses. Evolutionary reversals provide a means of escape by generating antigenic novelty and reflect fluctuating dynamics in immune responses, in which cross-immunity wanes, thereby controlling pathogen replication and facilitating virus transmission^30^. Escape mutations within or flanking functionally conserved epitopes come under selective pressure to revert to the wild type in hosts that do not mount an immune response against the epitope^43^. Amino acid reversions could also be a necessary consequence of a limited set of possible replacements at epitopes, a constraint on the repertoire of functionally viable amino acids^30^.

The Arg-180 substitution may have altered the positive charge of the G protein cysteine noose. The role of electrostatic charge in pathogenesis has been appreciated for HIV rapid progression to AIDS by the presence of charged residues at two amino acid positions on the gp120 subunit^44^. In Influenza A, several immune escape mutants showed an increased positive charge in HA and virions with a higher net positive charge in HA may promote favourable electrostatic interactions with target cells since host cell receptors possess a strong negative charge^45^. A change in surface charge (Glu to Lys) of the envelope glycoprotein was also implicated in the emergence of enzootic and epizootic viral phenotypes^46^. In addition, a potentially significant element of the immune escape repertoire is the selection of ‘adsorptive mutants’, showing enhanced binding to target cells^47^. An important aspect of receptor-binding avidity is that of electrostatic charge^45^. The Arginine replacement at position 180 may have resulted in increased net positive surface charge at the cysteine noose consequently promoting electrostatic interactions with target host cells. Future therapeutic design efforts will need to assess for possible loss of human G-directed mAbs cross-reactivity to epitopes containing the N178S and Q180R mutations.

Both pervasive positive selection and episodic diversifying selection were detected in amino acid positions within the sequenced G gene segment, six of which (227, 270, 287, 305, 306 and 313) were in agreement with previous studies^30,48^. Similarly, four putative N-glycan binding sites reported in this study (86, 144, 296, and 310) were previously reported for RSV G^49^. Amino acid position 144 was also identified in our study as being under positive selection by at least two methods.

In summary, our analyses show that the genotype BA dynamics in Kilifi have been marked by multiple co-circulating variants even within an epidemic, which are serially replaced over time perhaps by virus importations or evolution in situ. There has been gradual diversification specially in the C-terminal of the G protein possibly due to antibody-driven selection or functional constraints. In later epidemics (from 2007/8) the genotype was characterized by marked evolutionary reversions that reflect fluctuating immunological dynamics—either loss of pre-existing immunity or positive selection in a newly susceptible populations^30^—in the local communities.

Contemporary sampling and sequencing of RSVB particularly in Africa is still insufficient, compared to HIV-1 viruses, for which the regional spatial ecology has been extensively characterized^50,51^. Paucity of sequence data from neighboring countries limited our inferences on virus importations and movement within the sub-Saharan region. Future studies on local epidemics, transmission and spatial dynamics of the genotype BA could greatly benefit from improved sampling and sequencing in the region. Nonetheless, this study highlights the value of longitudinal sampling within a discrete location and of sequencing sufficiently over a long time to characterize RSV population dynamics. Further studies are required to determine the importance of variant-specific genetic differences in immune response and virus transmission. While the G gene is the fastest evolving RSV gene and has been extensively useful in RSV molecular epidemiology, studying the diversity of the rest of the RSV genome could allow for a deeper understanding of the long-term evolutionary dynamics of the RSV genotype BA.

## Methods

### Study samples and epidemic patterns

This study was part of longitudinal surveillance study established to understand the epidemiology and disease burden of RSV associated pneumonia^21^. The respiratory samples were collected into the universal transport media (UTM; Copan Diagnostics, USA). We used sequence data from RSV positive samples (nasal washes, aspirates and swabs) collected between January 2003 and August 2017. A majority of the samples were from children < 5 years admitted to Kilifi County Hospital (KCH) (2004–2017) with lower respiratory tract illness^20,21^. The 2003 samples were from the Kilifi RSV birth cohort acute respiratory infection cases^52^. All sample sets arise from the same catchment population and were processed similarly in the laboratory as detailed below.

Informed consent was sought from parents or guardians, and the study protocols were reviewed and approved by the Scientific and Ethical Review Unit, KEMRI, Kenya. All methods were carried out in accordance with relevant guidelines and regulations.

RSV epidemics in Kilifi typically start in November and run through to May with sporadic (inter-epidemic) infections occurring in June to October^21^. However, there is year-to-year variation and therefore we took a pragmatic approach and defined an epidemic as running from October of one year through to September of the following year. The dominant RSV group within a given epidemic was associated with ≥ 60% of the cases; otherwise, the groups were considered co-dominant.

### Viral detection, amplification and sequencing

RSV detection was performed by immunofluorescence antibody test (IFAT; RSV DFA kit, Light Diagnostics, UK). Beginning 2007, a real-time RT-PCR assay with primer/probe sets targeting the highly conserved nucleoprotein (N) gene was used to discriminate RSVA and RSVB for all respiratory samples collected during the surveillance period as previously described^24,53,54^. In 2015, the real-time RT-PCR assay for RSVB was updated following failed detection of drifted variants^55^. For genotyping purposes, a 900 base pair (bp) fragment of the G gene was amplified from purified viral nucleic acids of RSVB positive samples and sequenced in a nested PCR reaction as previously described^18,53^. Sequence assembly was done using Sequencher v5.0 (Gene Codes Corp., USA). The RSVB genotype BA viruses were identified following sequence alignments and phylogenetic analysis of the assembled sequences.

### Global data set

In addition to the Kilifi sequences, we retrieved RSVB G gene sequences collected between 2012 and 2017 from GenBank. We excluded sequences were shorter than 700 nucleotides, non-BA genotype sequences and those without sampling date or location information. Sequences were aligned using MAFFT 7.222 and alignments manually edited in Aliview^56^.

### Phylogenetic analyses

Model selection (ModelFinder) and Maximum likelihood (ML) phylogenetic trees were estimated with IQTREE 1.6^57,58^. Node support values were estimated using bootstrap resampling (1000 replicates). Temporal signal in the data was examined using TempEst^59^. Phylogenetic relationships and viral demographic histories were inferred using BEAST 1.10^60^. We used a GTR substitution model with discrete gamma distributed rate variation among sites and uncorrelated relaxed molecular clock with branch rates drawn form a lognormal distribution to account for evolutionary rate variation among lineages. A Skyride demographic prior with time-aware smoothing^61^ was selected and a CTMC rate reference prior was specified for the mean clock rate. Chain length of MCMC sampling was 300 million generations, sampling every 10,000. Stationarity and mixing were examined using Tracer 1.7 and maximum clade credibility (MCC) trees summarized using TreeAnnotator.

A variant was defined as a virus or group of viruses with 4 nucleotide differences compared to other viruses and/or falling into a distinct cluster with at least 60% bootstrap support^24^.

### Selection analysis

We tested the G gene data for evidence of natural selection using methods implemented in the Datamonkey 2.0 webserver^62^. Mean non-synonymous to synonymous rate ratio (d_N_/d_S_) was estimated using the SLAC method. Pervasive selection analyses were performed using SLAC and FEL methods. Episodic (frequently transient) selection was evaluated using MEME, and the FUBAR method was used to evaluate the difference between nonsynonymous and synonymous rates per codon site. Significance of SLAC, FEL and MEME results used a *P*-value cutoff of 0.05, and FUBAR results were assessed posterior probability of 0.9.

### N-linked glycosylation sites

The N-Glycosite web tool (http://www.hiv.lanl.gov/content/sequence/GLYCOSITE/glycosite.html) was used to identify putative N-glycosylation sites (amino acid configuration N-x-S/T, where x is not Proline (P)).

## Supplementary information

Supplementary figure.

Supplementary table.

## Data Availability

The Kilifi G gene sequences analysed here are available in GenBank (accession numbers KP862065-KP862529, KX775722-KX775849 and MH742792-MH742925). Other dataset used in the analysis are found in https://doi.org/10.7910/DVN/XJUA0Z.
