# Supplementary material for: Evolution of respiratory syncytial virus genotype BA in Kilifi, Kenya, 15 years on
Source: Sci Rep. 2020 Dec 3;10:21176. doi: 10.1038/s41598-020-78234-0 (PMC7712891; doi:10.1038/s41598-020-78234-0)

Evolution of Respiratory Syncytial Virus genotype BA in Kilifi, Kenya, 15 years on

Everlyn Kamau[a,‡,*], James R. Otieno[a,§], Clement S. Lewa[a], Anthony Mwema[a], Nickson Murunga[a], D. James Nokes[a,b] and Charles N. Agoti[a,c]

[a] Epidemiology and Demography Department, Kenya Medical Research Institute (KEMRI) – Wellcome Trust Research Programme, Kilifi, Kenya

[b] School of Life Sciences and Zeeman Institute (SBIDER), University of Warwick, Coventry, UK

[c] School of Health and Human Sciences, Pwani University, Kilifi, Kenya

‡ Present address: Nuffield Department of Medicine, University of Oxford, Oxford, UK

§ Present address: Fogarty International Center, NIH, Bethesda, MD, USA

[*] everlyn.kamau@ndm.ox.ac.uk

Supplementary Figure S1 Site-wise differences between the non-synonymous (β) and synonymous (α) substitution rates in the analyzed G glycoprotein region.

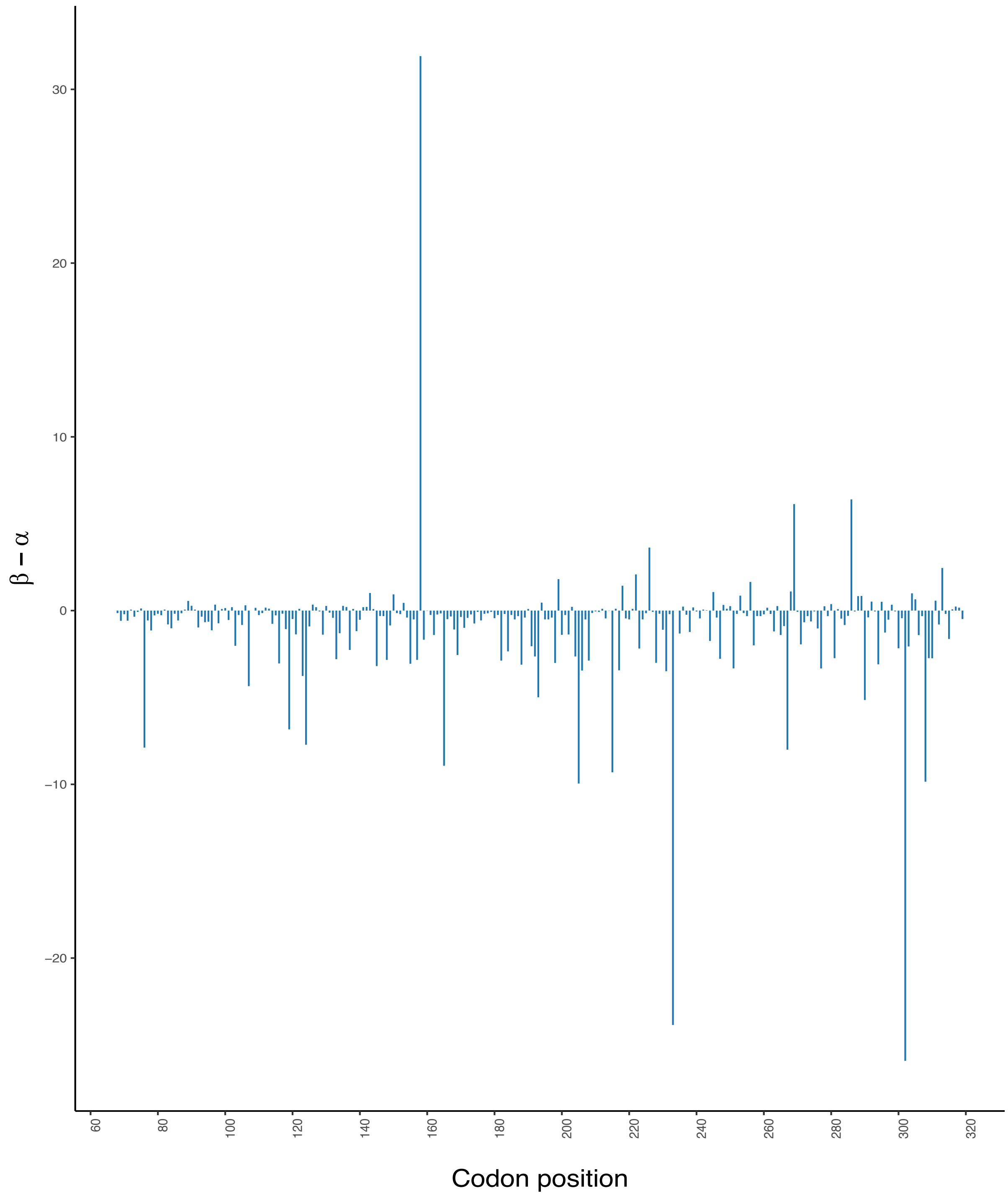

Supplement: Supplementary file 1 — Supplementary figure. [file 41598_2020_78234_MOESM1_ESM.pdf]
