# Supplementary material for: Evolution of respiratory syncytial virus genotype BA in Kilifi, Kenya, 15 years on
Source: Sci Rep. 2020 Dec 3;10:21176. doi: 10.1038/s41598-020-78234-0 (PMC7712891; doi:10.1038/s41598-020-78234-0)
Supplement: Supplementary file 2 — Supplementary table. [file 41598_2020_78234_MOESM2_ESM.docx]

Supplementary Table 1. Yearly distribution of RSV cases by age.

| Year | 0-12 months | 13-24 months | 25-36 months | 37-48 months | 49-60 months |
| --- | --- | --- | --- | --- | --- |
| 2002 | 91 | 11 | 3 | 2 | 1 |
| 2003 | 57 | 11 | 3 | 1 | 0 |
| 2004 | 133 | 21 | 12 | 6 | 0 |
| 2005 | 102 | 15 | 3 | 4 | 0 |
| 2006 | 166 | 24 | 16 | 2 | 2 |
| 2007 | 196 | 29 | 9 | 1 | 0 |
| 2008 | 144 | 22 | 6 | 7 | 2 |
| 2009 | 162 | 29 | 8 | 5 | 2 |
| 2010 | 199 | 47 | 10 | 2 | 1 |
| 2011 | 197 | 24 | 9 | 4 | 0 |
| 2012 | 183 | 34 | 13 | 4 | 1 |
| 2013 | 74 | 12 | 1 | 1 | 0 |
| 2014 | 159 | 10 | 7 | 4 | 0 |
| 2015 | 119 | 12 | 6 | 2 | 0 |
| 2016 | 85 | 7 | 3 | 1 | 1 |
| TOTAL | 2067 | 308 | 109 | 46 | 10 |
